# Compensation System for Biomagnetic Measurements with Optically Pumped Magnetometers inside a Magnetically Shielded Room

**DOI:** 10.3390/s20164563

**Published:** 2020-08-14

**Authors:** Anna Jodko-Władzińska, Krzysztof Wildner, Tadeusz Pałko, Michał Władziński

**Affiliations:** Warsaw University of Technology, Faculty of Mechatronics, Institute of Metrology and Biomedical Engineering, Boboli 8 St, 02-525 Warsaw, Poland; krzysztof.wildner@pw.edu.pl (K.W.); tadeusz.palko@pw.edu.pl (T.P.); michal.wladzinski@pw.edu.pl (M.W.)

**Keywords:** optically pumped magnetometer, magnetically shielded room, Helmholtz coils, biomagnetism

## Abstract

Magnetography with superconducting quantum interference device (SQUID) sensor arrays is a well-established technique for measuring subtle magnetic fields generated by physiological phenomena in the human body. Unfortunately, the SQUID-based systems have some limitations related to the need to cool them down with liquid helium. The room-temperature alternatives for SQUIDs are optically pumped magnetometers (OPM) operating in spin exchange relaxation-free (SERF) regime, which require a very low ambient magnetic field. The most common two-layer magnetically shielded rooms (MSR) with residual magnetic field of 50 nT may not be sufficiently magnetically attenuated and additional compensation of external magnetic field is required. A cost-efficient compensation system based on square Helmholtz coils was designed and successfully used for preliminary measurements with commercially available zero-field OPM. The presented setup can reduce the static ambient magnetic field inside a magnetically shielded room, which improves the usability of OPMs by providing a proper environment for them to operate, independent of initial conditions in MSR.

## 1. Introduction

Electrophysiological phenomena in the human body lead to the occurrence of magnetic field [1]. While electric biosignals are affected by the presence of insulating layers (fat, bones) and varied conductivity of the body tissues, magnetic field can easily penetrate through. Contactless measurements using magnetometers can avoid difficulties related to the electrode’s attachment to the skin.

Measurements of the fields generated by the ionic currents flowing through the fibers of the cardiac muscle (magnetocardiography (MCG) [2]), activity of the cerebral cortex neurons (magnetoencephalography (MEG) [3]) or detection of iron stores (liver susceptometry [4]) are already implemented in clinical practice [5,6,7]. Magnetism of neural system (magnetoneurography (MNG) [8]), lungs (magnetopneumography [9]), stomach (magnetogastrography (MGG) [10]) and intestine (magnetoenterography (MENG) [11]) is an additional topic of research [12]. Study on fetal development (fetal magnetocardiography (fMCG) [13], fetal magnetoencephalography (fMEG) [14]) is another specific application of magnetic sensors. The vernix caseosa surrounding the fetus in the second half of pregnancy has low electrical conductivity, which significantly favours magnetography over electrography [15].

Magnetic fields generated by living organisms are of extremely low amplitude (fT – pT). Biomagnetic measurements can be thus affected by much higher electromagnetic noise or disturbing fields, e.g., Earth’s magnetic field (50 µT). Comparison of these values shows how challengeable biomagnetometry is and how important the sensitivity of sensors and proper magnetic shieldings are.

Measurements of biomagnetic fields are commonly done using superconducting quantum interference device (SQUID) sensor arrays. They are the most sensitive known magnetometers and can detect extremely subtle magnetic fields [16]. The first magnetocardiogram and magnetoencephalogram were measured using SQUIDs in 1970 and 1972, respectively [17,18]. Unfortunately, the SQUID-based systems have some limitations. They are expensive in use as sensors have to be cooled down close to absolute zero. The distance between a subject and the sensor is significant due to the dewar presence and no geometric adaptability to match subject’s anatomy is provided. The higher the sensor-to-signal source distance, the lower the amplitude of detected magnetic field.

In the last two decades, the optical magnetometry has seen a rapid progress, offering a room-temperature alternative for SQUIDs: optically pumped magnetometers (OPM). Although atomic sensors have been already used for measuring human magnetic fields in the late 1970s, they could not compete with SQUIDs [19]. In the beginning of the new century, few groups came back to the idea of uncooled sensors [20,21,22,23], reaching sensitivities comparable [24], or even better [25], than SQUIDs can provide. Nevertheless, it was the miniaturization of new magnetometers, which made them a new player in biomagnetism [26].

Flexible optical and electrical wiring of OPMs, together with a small size sensor head, enable minimizing the sensor-signal source distance, which leads to higher amplitudes of received signal [27,28]. OPMs have been successfully used in magnetocardiography [29], magnetoencephalography [30,31], magnetomyography [32] and fetal magnetocardiography [33]. Furthermore, new possibilities in biomagnetometry are enabled, as sensors can be easily attached to subject’s body, e.g., using dedicated anatomy-adapted 3D printed holders. This feature is expected to make a special impact while working with patients, whose movements are difficult to control (e.g., children, patients with Parkinson’s disease). Moving magnetoencephalography [34] and exercise magnetocardiography [35] with OPMs were proven to be possible.

The SERF OPMs require a near zero ambient magnetic field to operate [36,37], which cannot be easily achieved by a standard magnetic shielding. The shielding factor of magnetically shielded rooms (MSR) is defined by the number of the damping layers, the frequency, the permeability and the dimensions of the chamber. While magnetically shielded portable forehand-size chambers have a satisfying shielding-to-volume ratio and provide a magnetic field low enough for OPMs to operate [38], the problem may occur when using a man-size MSR. In a two-layer magnetically shielded room (number of µ-metal layers), a static magnetic field of ~50 nT is achieved [39]. This value of residual magnetic field could be compensated by built-in internal compensation coils of zero-field magnetometer. However, for commercially available QuSpin Zero-Field Magnetometers Gen-1 (QZFM, QuSpin Inc., Louisville, KY, USA) [40], which can cancel residual static fields up to 50 nT, the limit value of magnetic induction that can be cancelled is achieved [36]. Standard MSRs may be not sufficiently shielded to provide a low enough residual field for OPMs to operate and an additional compensation is needed. Two-layer magnetically shielded rooms are common worldwide as SQUIDs do not require an ambient magnetic field as low as SERF OPMs do. Adaptation of these rooms to the new technology can be made.

Static magnetic fields can be cancelled with coil systems providing uniform magnetic fields [41]. If dynamic changes in magnetic fields inside MSR affect measurements, a dynamic compensation can be used [42,43]. Even moving magnetoencephalography with OPMs was proven to be possible when complex bi-planar coils setup for nulling the magnetic field and a magnetic field gradient was engaged [44].

While the theoretical basis of the residual field compensation is known and advanced compensating systems are commercially available [45,46,47], we present a customized design to the certain application: working with OPMs inside MSR. The presented cost-efficient battery-powered static field compensation system, based on the Helmholtz square coils can be easily installed inside MSR and its total cost does not exceed 200 Euros. The system is used to bring OPMs to their operational range, when the initial conditions in the shielded room are not sufficient.

## 2. Materials and Methods

**Figure 1:**
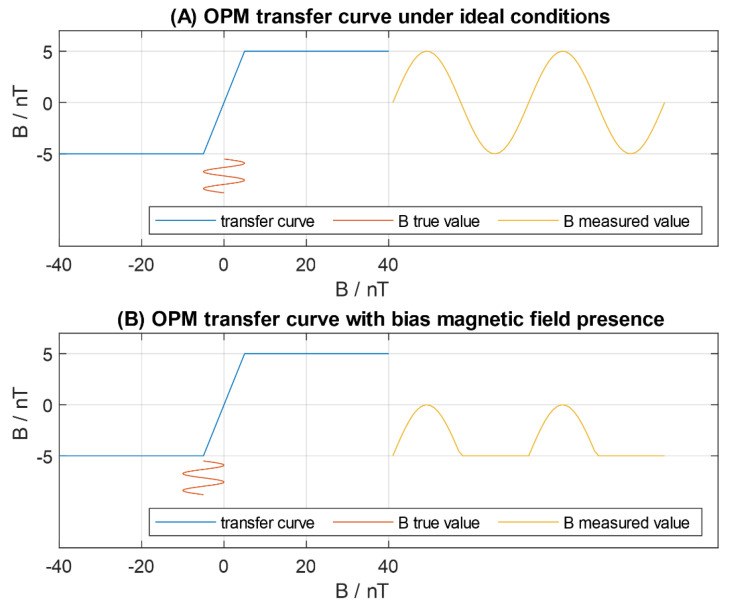
Conceptual transfer curve of the zero-field optically pumped magnetometer electronic module output: (**A**) under ideal conditions; (B) with the presence of the bias magnetic field. In (**B**), signal saturation is visible.

Zero-field optically pumped magnetometers require a close to zero residual magnetic field. In commercially available OPMs from QuSpin (QZFM), three-axis integrated field cancellation coils are installed around the vapour cell and provide a field zeroing procedure [36]. The procedure is done automatically with the dedicated software at the beginning of measurements. The first generation (Gen-1) of QZFM can cancel the residual magnetic field of 50 nT, and thus an ambient magnetic field below this value is required for OPMs to operate. The measurement dynamic range of SERF OPMs is defined by the zero-field resonance and may differ depending on a manufacturer. In order to operate the SERF OPM in its linear regime, the dynamic range must be significantly smaller than the magnetic resonance width [48]. Full width at half maximum (FWHM) of the zero-field resonance in QuSpin OPMs (QZFM Gen-1) is ~30 nT and electronic control unit output provides a measurement dynamic range of ± 5 nT after zeroing procedure [40]. The conceptual transfer curve of the sensor in the operating mode is presented in Figure 1A.

Unfortunately, ideal conditions are not easy to achieve in a man-size two-layer shielding room. There are few reasons for that. Firstly, an ambient magnetic field in the measurement volume can be initially higher than 50 nT. In this situation, the magnetic field must be compensated to make the sensor field zeroing procedure possible. Secondly, even after field zeroing process, the operating point can be close to the limit of compensation capabilities. Finally, the magnetic field can change during the measurement more than ±5 nT dynamic range of the OPM, especially in long lasting sessions, and the operating point chosen initially may not be adequate anymore. These conditions, assuming that the OPM can still operate, may lead to sensor overdrive and signal saturation, presented in Figure 1B.

To overcome difficulties that may appear during measurements using zero-field magnetometers, we decided to design a three axis compensation coils system. The system was supposed to be installed inside the magnetically shielded room located at Warsaw University of Technology, Faculty of Mechatronics. The MSR shell consists of two layers of µ-metal and one layer of aluminium between them and has inner dimensions of 4.0 × 3.0 × 2.4 [m] (Vacoshield Advanced, Vacuumschmelze GmbH and Co. KG, Hanau, Germany [49]). The MSR in our facility allows for static shielding of the magnetic field only. It does not incorporate built-in compensating coils, so an active shielding is not possible without an additional compensating system. The attenuation level of the static magnetic field equals 54 dB, for the 0.01 Hz it is 32 dB, and for 0.1 Hz its value is at the level of 38 dB.

The Helmholtz coil, consisting of two identical coils, can produce a region of uniform magnetic field and thus can be used for cancelling an ambient magnetic field. A Helmholtz pair can be both circle and square. In both cases, coils with an equal number of turns are placed coaxially. The coils are connected in series, which provides the same electric current flow through both of them. The direction of the electrical current in the coils is set to generate the same direction of magnetic flux. Helmholtz spacing, a distance between the coils providing the homogeneity of the field, is equal to the radius of the coil for circle coils and equal to 0.5445 times the length of a side for square coils [50]. The uniformity of the field at the central volume of the coils is reached at the expense of variation in field strength at a distance from the centre. If slight nonuniformity at the centre is acceptable, different spacings are used to obtain an increase in the length of the quasi-uniform field [50]. Changing the coils spacing to, e.g., 1.15 times the Helmholtz spacing would result in an increase in the side-length of the quasi-uniform field volume from 20% to 60% of the coils’ side length, at the expense of the magnetic field uniformity [50]. In this case, the magnetic field at the distance (measured from the centre of the quasi-uniform field volume) of 20% of the coils’ side length would be ~2% higher than the field measured in the centre. However, the magnetic field at the distance of 30% of the coils side length would be ~2% lower than the field measured in the centre. In other words, across the above-mentioned distance equal 10% of the coils’ side-length, gradient will be equal to ~4% of the magnetic field measured in the centre of the quasi-uniform field volume. For 50 nT (the expected magnitude of the static magnetic field to be cancelled with the compensation system), this would result in a ~2 nT (~4% of 50 nT) change across 20 cm (as 20 cm is 10% of designed 2 m square coils’ side-length), which is equal ~10 pT/mm. The magnetic field gradient in a two-layer MSR is typically lower than 30 pT/mm [39], and OPM requires a gradient not higher than 10 nT/3 mm = 3.3 nT/mm to operate properly. Thus, theoretically, even with the coil spacing providing less uniform magnetic field than in case of the Helmholtz spacing, the gradient produced by compensation coils should not deteriorate OPM performance significantly. Nevertheless, we decided to use the Helmholtz spacing in our system.

**Figure 2:**
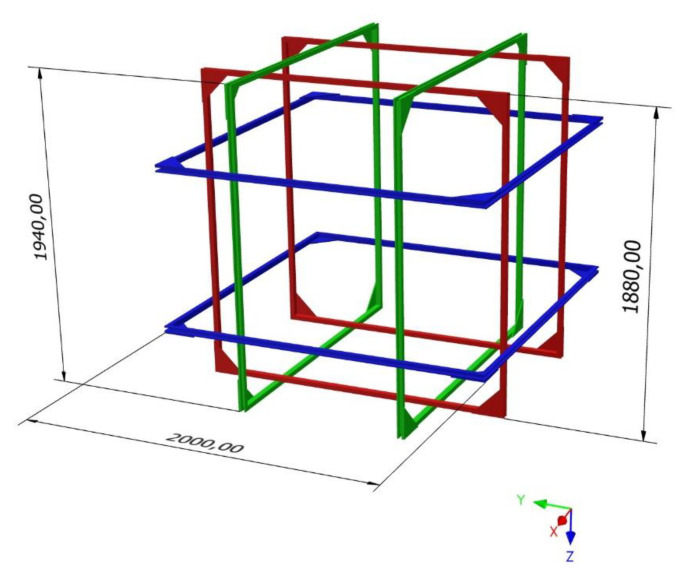
CAD model of the designed compensation system, based on square Helmholtz coils; dimensions in millimeters. Figure created using Inventor^®^ CAD software (Autodesk, Inc., San Rafael, CA, USA [53]).

Square Helmholtz pair provides better uniformity of magnetic field in comparison to circular coils [51]. Furthermore, their shape is more useful in practice: the construction can stand alone and can be easily assembled and disassembled. The CAD model of the designed 3D Helmholtz coil system is presented in Figure 2. The CAD model was created using open-source Free CAD software [52].

**Figure 3:**
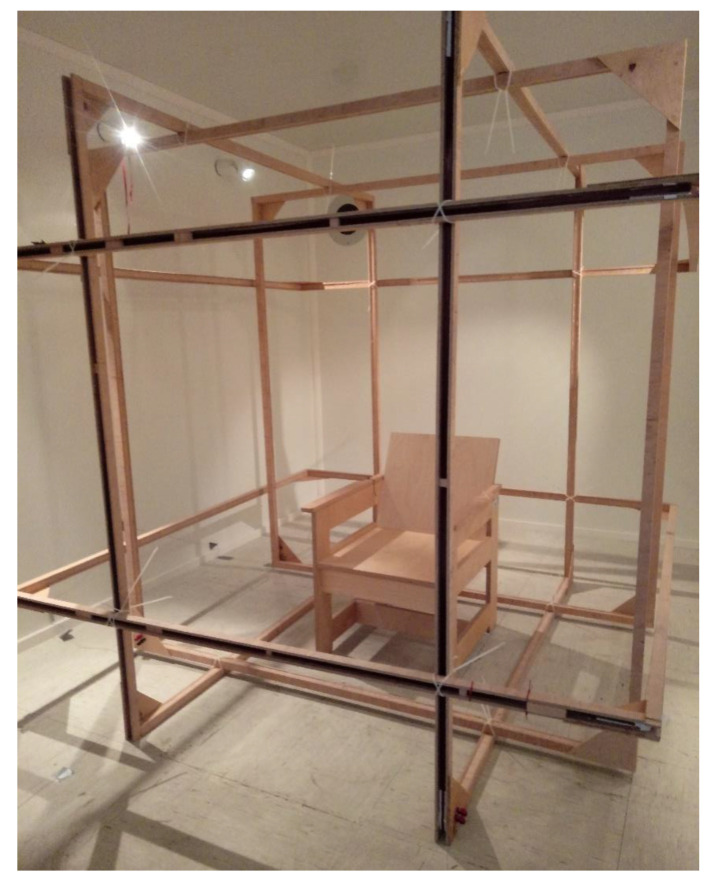
Wooden Helmholtz coils setup installed in a two-layer magnetically shielded room (Vacoshield Advanced, Vacuumschmelze GmbH & Co. KG, Hanau, Germany) at Warsaw University of Technology, Faculty of Mechatronics.

For biomagnetic measurements (e.g., magnetoencephalography, magnetocardiography) a large volume of uniform magnetic field is required. Supporting system for the triaxial compensation coils was made of wood with 2-m-length of a side (Figure 3). The size was limited by the dimensions of the entrance to the magnetically shielding room, but is still big enough for a human subject and can be easily accessed. The volume with the magnetic field homogeneity better than 0.1% is about 1/5 of the entire length [51], which provides a uniform volume with the side length equal to 0.4 m. Significant dimensions help to avoid the situation when movements of the subject could have led to touching of the coils frame, inducing variations in magnetic field generated by the compensating system. Each coil was wound with 30 and 60 turns of enameled wire independently. Such an approach brings some flexibility in terms of the range of the magnetic field it is possible to generate. The 30-turn windings (with higher wire cross-section area) could be used to work with higher currents or the 60-turn windings (with smaller wire cross-section area) while working with smaller currents. It is also possible to connect windings in series to obtain 90-turn windings. Double winding also gives the possibility to use one set of coils to compensate the residual magnetic field and the other one to introduce external signals. It is planned to use such a configuration in further research.

The 30-turn coils were made with 1-mm^2^ cross-section area copper wire, resulting in a maximum resistance equal to 6 Ω per coil. This enables a high current flow of up to 1 A using 12 V power supply. The 60-turn coils were made with 0.1-mm^2^ cross-section area wire and gave maximum resistance of 150 Ω per coil.

**Figure 4:**
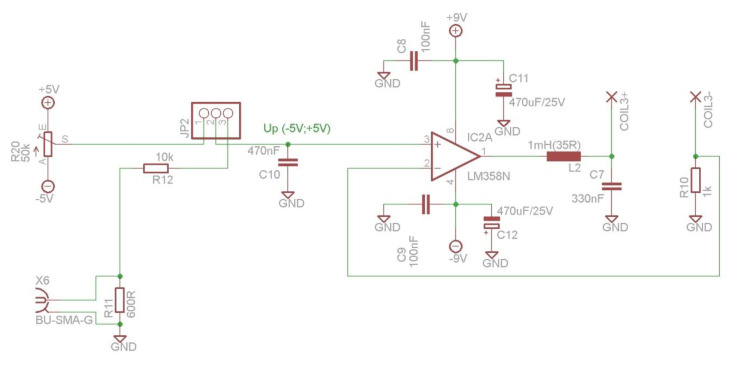
Electrical circuit of the current source for one pair of compensation coils, where BU-SMA-G—a female SMA connector for voltage control signal, JP2—jumper to choose the method of controlling the current source (manually via potentiometer or voltage control, e.g., via acquisition card).

Compensation coils have to be driven with a stable current source. The dedicated circuit, based on a Howland current source, was designed and is presented in Figure 4. The source is battery-powered and provides filtration of the current supplying the coils to ensure current stability. The battery supply was used to avoid introducing unwanted noise from mains. The whole compensation system requires rather small current (20 mA), hence, while powered with two 9 V batteries, it can last for reasonably long time (~10 h). The current value can be controlled manually via potentiometer or voltage control (e.g., via acquisition card). The output may be set in the range from −5 to 5 mA separately for each of three pairs of coils, resulting in an expected magnetic field strength of ~500 nT at the center of Helmholtz coils, when using 90-turn windings.

The current source provided stable current values with less than 0.1 µA deviation from set parameters. During the 45-min test, the current drifted by ΔI = 0.51 µA, which corresponds to the changes of the magnetic field ΔB ~33 pT. The obtained accuracy provided a stable value of magnitude of the magnetic field generated by the coils. Recordings of the current flowing through the coils and the analog voltage output of the fluxgate magnetometer Fluxmaster (Stefan Mayer Instruments GmbH & Co. KG, Dinslaken, Deutschland [54]) were taken with 2 Keithley Fluke 8845A Precision Digital Multimeter (Fluke Corporation, Everett, WA, USA [55]) at a sample rate equal to 5 S/s.

The system was designed to change the bias magnetic field in all directions in the range of ±500 nT. The QuSpin OPMs Gen-1 provide the compensation of an ambient magnetic field up to 50 nT. The obtained range of the magnetic field adjustment gave a large margin for measurements, exceeding the field zeroing capabilities of OPMs.

To acquire the data from QuSpin sensors the NI-USB(BNC) 6218 DAQ (National Instruments Corporation, Austin, TX, USA [56]) card was used together with the dedicated application developed in LabView^®^ software. The raw data, four signals from two QuSpin OPM sensors representing the magnetic field in two orthogonal axes (Z and Y axes measured simultaneously), were sampled at 500 S/s with 16-bit resolution and streamed to the binary file (in big-endian order). The data stored in the file contain only fundamental information: sampling frequency (double, 8 Bytes), number of channels (single, 4 Bytes), data length (single, 4 Bytes) and finally the QuSpin data (double, 8 Bytes per sample, without multiplexing). The application, however, was provided with additional functionalities: real time power spectrum monitor, acquisition path evaluation (via signal power and signal to noise ratio calculation while testing signal applied), simple preview of signals and a timer which could set the recording time and to visualize the time elapsed. The power spectrum monitor was also accompanied by a set of filters to choose from, which could remove the mains and its harmonics, the residual hum of the data acquisition card and the DC offset. This was intended to evaluate possibilities of signal quality improvement before the final recording. Only the raw data were recorded to preserve the data format simple and flexible in terms of further processing.

## 3. Results

### 3.1. Compensation System Characterisation

The designed and built system was installed inside the two-layer magnetically shielded room at Warsaw University of Technology, Faculty of Mechatronics. To investigate the relationship between the magnetic induction generated by the Helmholtz coils and the current flow in wires, the fluxgate magnetometer Fluxmaster with measurement range of ±2 µT and 0.1 nT resolution was used [54]. The sensor was located in the centre of the setup and directed perpendicularly to each of three coils planes. The coils were supplied with the current in the range from −2.0 to 2.0 mA, which provided bias magnetic field changes in the range of ± 100 nT produced using only one set of coils (60 turns).

The relation of the current value vs. the value of the magnetic field was investigated. Measurements were carried out as follows: the current flowing through the compensation coils was changed in such a way as to set magnetic field inside measurement volume at predefined levels: from −100 to 100 nT in 10 nT steps. First the direction of changes was ascending, from −100 to 100 nT, then descending, from 100 to −100 nT. This procedure was repeated two times, giving four datapoints for each interval in total.

As the sensor may have a slight dependence on direction, the measurements were performed in two directions, positive and negative, with respect to the magnetic field produced by the compensation coils. By positive, we assume the sensor oriented in the direction of the magnetic field vector; negative relates to the reversed setting.

**Figure 5:**
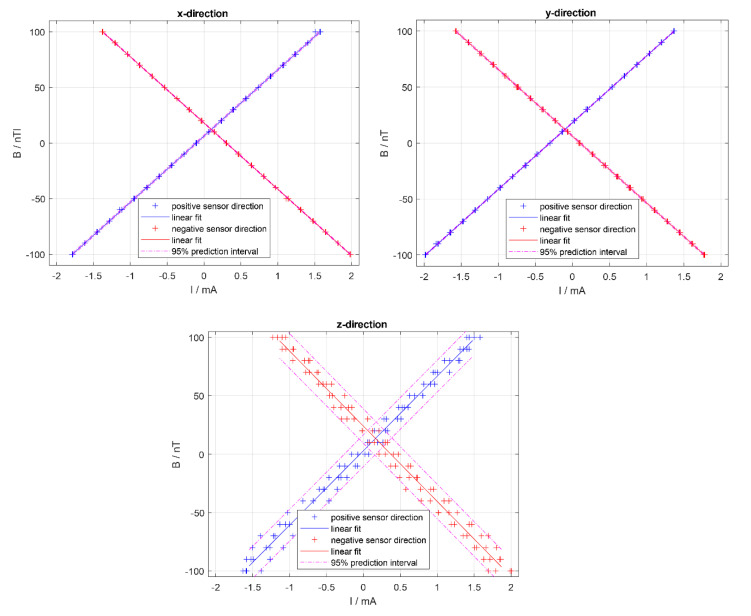
Current supplying the coils vs. the magnetic field generated in the central volume; blue plot—measurements in the positive sensor direction, red plot—measurements in the negative direction, where positive relates to the same direction as the magnetic field vector and negative relates to the opposite. For both series of data, linear fit was calculated, as well as 95% prediction interval. Measurement directions (x, y, z) correspond to the coordinate system in Figure 2.

Results for each of three Helmholtz coils are presented in Figure 5. Measurements directions (x, y, z) correspond to the coordinate system in Figure 2.

Linear regression calculated using Matlab^TM^ (The MathWorks, Inc., Natick, MA, USA [57]) polyfit function gave equation B[nT] = p_1_ I[mA] + p_2_, where p_1_ and p_2_ are the polynomial coefficients and I[mA] is the current feeding the coils. Linear regression coefficients in descending powers (p_1_ and p_2_) with a standard error (σp1 and σp2) and determination coefficients R^2^ calculated for the measurements done in each direction are presented in Table 1.

Approximate value of the residual magnetic field in the centre of the magnetically shielded room was determined from the linear fit of data. The calculation of the relation between the current and the magnetic field produced gave the value of the magnetic field ordinate for the current value set to zero. This value is approximately equal to the residual magnetic induction within the magnetically shielded room. The residual values for all three directions are as follows: x_max_ ~18 nT, y_max_ ~18 nT, z_max_ ~24 nT. The magnetic field variations were highest in the vertical (z) direction, reaching −30 nT on the other days.

### 3.2. Preliminary Measurements

**Figure 6:**
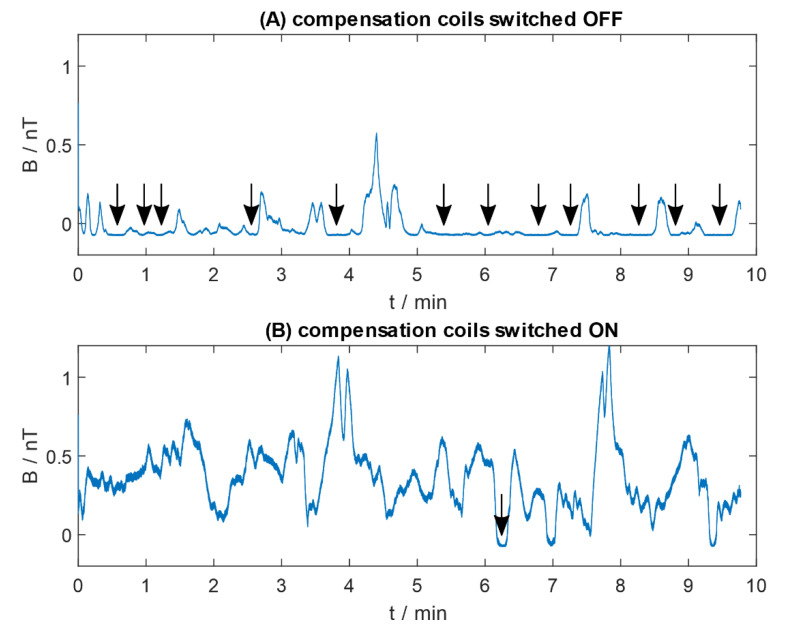
Residual magnetic field measured with QuSpin zero field magnetometer inside two-layer magnetically shielded room with the additional compensation coils: (**A**) switched off; (**B**) switched on. Arrows indicate the areas of signal saturation.

Preliminary measurements of the ability to compensate the magnetic field in the volume of interest with QuSpin zero-field magnetometers were made (Figure 6). The optically pumped magnetometer was located in the centre of the compensation coils setup, perpendicularly to the plane of the green coils (−y direction) in Figure 2 (left and right vertical coils in Figure 3). Initially, measurements of the magnetic field were done without any additional active shielding (compensation coils switched off). Subsequently, the coils were switched on and the magnetic field was minimized manually, based on the magnetic induction values obtained with the fluxgate magnetometer Fluxmaster. The measurements of the magnetic field using OPM were performed again, with the compensation coils switched on.

Even if the sensor was calibrated properly with the dedicated QuSpin software at the beginning of each measurement, the magnetic field induction measured without additional compensation shows multiple ‘flat’ areas (indicated in the picture with arrows, Figure 6A). In these areas, the magnetic field entering the centre of MSR is expected to be outside the compensation capabilities of used OPM. Use of the additional compensation coils changed the bias magnetic field, and signal saturation occurred only once.

The amplitudes of the residual magnetic field changes in the measurements done with and without additional active compensation (compensation coils switched on and off, respectively) cannot be compared, as the signal was saturated. Nevertheless, as no additional signal source was present inside MSR, the amplitude was expected to be similar. The difference is a result of shifted bias magnetic field.

**Figure 7:**
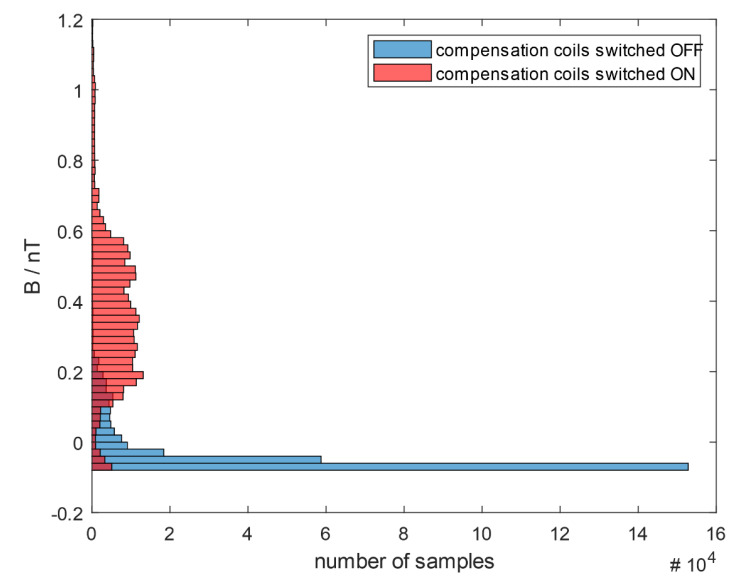
Distribution of magnetic induction of residual magnetic field measured with QuSpin zero field magnetometer inside the two-layer magnetically shielded room with the additional compensation coils switched off (blue) and switched on (red). The bin width was set to 0.02 nT, the number of bins: 66 (red) and 43 (blue). The skewed histogram of the measurement taken with the coils switched off (blue) with extreme dominant number of samples of magnetic induction in the range from −0.08 to −0.06 nT corresponds to the signal saturation.

To investigate the distribution of the measured residual magnetic field, histograms for both data series from Figure 6 were done (Figure 7). A bin width was set to 0.02 nT, which resulted in 66 bins for measurement done with the active shielding and 43 bins, when the coils were switched off. The number of bins already suggest a different distribution of the magnetic field. Histogram of measurement taken with the coils switched off (blue) is skewed with extreme dominant number of samples of the magnetic induction in the range from −0.08 to −0.06 nT. This corresponds to the areas of signal saturation.

Histogram of data obtained with the additional compensation (red) is much more symmetric compared to the previous one (blue). This proves that the magnetic induction was distributed in the full range of OPM used. The sensor overdrive did not significantly influence the measurement and the data could be used for further analysis.

Comparison of datasets obtained with and without the additional compensation of magnetic field, indicate no usability of data measured without compensation system switched on in magnetography of human magnetic fields.

It is expected that the higher the current feeding the coils, the more magnetic field noise originating from the current supply. The noise inside MSR at Faculty of Mechatronics, WUT measured with QuSpin OPM in the band 3–100 Hz was as high as 1.5 pT/√Hz for frequencies near DC, down to 50 fT/√Hz for frequencies above 70 Hz on the day the preliminary measurements were taken. For frequencies below 10 Hz, the noise observed with the compensation system turned on was on the same level, or even lower, as when the system was turned off. For higher frequencies additional compensation resulted in the additional noise, whose level was significantly higher, reaching −300–400 fT/√Hz in the worst case. Changing the ambient magnetic field inside MSR to make OPMs operational and the measurement possible is at the expense of a higher noise level.

## 4. Discussion

For proper operation, spin exchange relaxation-free, optically pumped magnetometers require a low magnetic field environment. Commercially available OPMs from QuSpin (QZFM Gen-1) can cancel an ambient magnetic field of magnetic induction below 50 nT, but this value is not easily achieved in the most common two-layer magnetically shielded rooms. The surroundings and maintenance of the MSR have a significant impact on the measurement and may lead to sensor overdrive and signal saturation.

The designed triaxial compensation system, based on square Helmholtz coils, enables generation of the magnetic field in the range from −500 to +500 nT, when supplied from the dedicated current source with the current in the range from −5 to 5 mA. The system was used to change the ambient magnetic field inside the magnetically shielded room and made measurements with OPMs possible, independently from the magnetic field variations. The presented setup improves the usability of zero field optically pumped magnetometers in standard MSR. The proposed solution can be used for decreasing an ambient magnetic field inside a magnetically shielded room to achieve a better environment for OPMs operation. While the presented setup was designed for commercially available QuSpin zero-field magnetometers (Gen-1) it could be extended to use with different OPMs operating in SERF regime to move them to their operational range. When OPMs’ built-in cancelling coils are not capable to handle the residual magnetic field within a poorly shielded environment, our compensation system is capable to null up to few hundred nT of the static magnetic field to make OPMs operable. The introduced cost-efficient system can be used for the adaptation of two-layer MSRs to the new technology.

Preliminary results show an improvement in measurements using QZFM optically pumped magnetometers. A compensation of the static residual field of the shielded room with developed setup was possible and successfully used to overcome sensor overdrive. Cancelling an ambient magnetic field inside MSR, in order to make OPMs operational and the measurement possible, was at the expense of an increase in the noise level. The noise in the presented solution is non-negligible and an additional technique should be applied to perform biomagnetic measurements. The technique applied should depend on the application. Averaging, which is often used for evoked magnetoencephalography, is a common solution in recording low energy signals generated by living subjects. In other cases, filters should be applied, but again, this could be insufficient for a simple waveform recording. With two sensors, the measurement could be performed in a gradiometer configuration or an additional sensor could be used as a reference in an adaptive filtering technique. The size of the compensation system developed provides a uniform magnetic field in a volume with a side length equal to 0.4 m. The proposed construction is suitable for measurements of biomagnetic fields of human subjects.

Nevertheless, the manual control of the magnetic field compensation may be insufficient in long measuring sessions (e.g., evoked fields). Two-layer MSR provides a 32–38 dB attenuation of an external magnetic field for a frequency range from 0.01 to 0.1 Hz. A slowly changing magnetic field entering the magnetically shielded room may cause signal saturation, and even the working point could leave the range of the built-in OPM compensation. These problems could be avoided by using an automatic compensation system, which is a field of further development.

## Figures and Tables

**Table 1:** Linear regression coefficients in descending powers (p1 and p2) with a standard error (σp_1_ and σp_2_) and determination coefficients R2 of linear regression B[nT] = p1 I[mA] + p2 calculated for the measurements done in each direction.

Direction	p_1_	σp_1_	p_2_	σp_2_	R^2^
x	59.5817	0.0571	6.3455	0.0583	1
−x	−59.5783	0.0298	18.0487	0.0316	1
y	59.6837	0.0351	18.2497	0.0372	1
−y	−59.7227	0.0457	6.1152	0.0466	1
z	63.7962	0.7783	3.0129	0.7352	0.988
−z	−64.4221	0.8562	23.9336	0.8600	0.986
